# Anthropometric measures and the risk of developing atrial fibrillation: a Swedish Cohort Study

**DOI:** 10.1186/s12872-021-02415-6

**Published:** 2021-12-18

**Authors:** Isac Zia, Linda Johnson, Ensieh Memarian, Yan Borné, Gunnar Engström

**Affiliations:** Department of Clinical Sciences, IKVM, Lund’s University, Jan Waldenströms gata 35, CRC, House 60, 13th Floor, 205 02 Malmö, Sweden

**Keywords:** Atrial fibrillation, Obesity, Hazards ratio, Anthropometric measures, BMI

## Abstract

**Aims:**

Obesity is a risk factor for several cardiovascular diseases (CVDs), including atrial fibrillation (AF). However, it is less clear whether overall fat or abdominal fat distribution are most important for risk of developing AF. This study investigates how different anthropometric measures correlate to the risk of developing clinical AF in the Malmö Diet and Cancer cohort (MDC-cohort).

**Methods:**

The MDC-cohort (n = 25,961) was examined in 1991–1996. The endpoint was clinical AF diagnosed in a hospital setting, and retrieved via linkage with national registers. Hazard Ratios (HR) for incident AF was calculated in relation to quartiles of body mass index (BMI), waist circumference, waist hip ratio, waist height ratio, body fat percentage, weight and height, using Cox regression with adjustment for age, biological (e.g. blood pressure, diabetes, blood lipid levels), and socioeconomic risk factors.

**Results:**

After adjustment for multiple risk factors, the risk of AF was significantly increased in the 4th versus 1st quartile of weight (HR for men/women = 2.02/1.93), BMI (HR = 1.62/1.52), waist circumference (HR = 1.67/1.63), waist to hip ratio (HR = 1.30/1.24), waist to height ratio (1.37/1.39) and body fat percentage (HR = 1.21/1.45) in men/women. Measures of overall weight (BMI, weight) were slightly more predictive than measures of abdominal obesity (waist hip ratio and waist height ratio) both in men and women.

**Conclusion:**

All measures of obesity were associated with increased risk of developing AF. Both overall obesity and abdominal obesity were related to incidence of AF in this population-based study, although the relationship for overall obesity was stronger.

**Supplementary Information:**

The online version contains supplementary material available at 10.1186/s12872-021-02415-6.

## Introduction

Obesity is an established risk factor for several cardiovascular diseases (CVDs) [1–4], including increased incidence of atrial fibrillation (AF) [5–8]. Obese individuals have a 50% increased risk of developing AF [5] and it has been estimated that a one-unit increase of mean BMI in the population could result in 6–8% more patients with AF [9]. Considering that the prevalence of obesity is increasing [10, 11], it is likely that prevalence of obesity-related AF will increase as well [12].

Obesity increases total blood volume, which causes structural changes to the heart, such as left and right ventricular hypertrophy [13–15]. Additionally, it leads to increased epi- and pericardial adipose tissue [16], which is associated with higher AF recurrence rate after ablation and higher burden of symptoms in patients with AF [6]. These structural changes alter cardiac electrical circuits, which could increase the risk of AF [13–16].

Fat distribution is of importance in the risk assessment for several cardiometabolic diseases, and it is widely accepted that the prognostic significance of increased intra-abdominal fat, such as increased waist circumference (*WC*) or waist to height ratio (*WHtR*), is more disadvantageous than increased subcutaneous fat [11, 17–19]. However, the role of abdominal fat distribution is less clear with respect to incidence of AF. Measures of *overall* obesity (i.e. body mass index *(BMI)*, body fat percentage *(BF %)* and weight) and *abdominal* obesity (i.e. WC, waist to hip ratio (*WHR*) and WHtR) have both been shown to correlate positively with the risk of developing AF [4, 5, 8, 9, 12, 20, 21]. This raises the question if the role of intra-abdominal fat distribution is similar for AF as compared to other cardiometabolic diseases.

The purpose of this study was to investigate the relationship between different anthropometric measures (BMI, weight, height, WC; WHR, WHtR and BF %) and incidence of AF in a population-based cohort study, to further investigate the relationship between fat distribution and AF in a large population-based cohort study with long follow-up.

## Methods

### Study population

Data from the “Malmö Diet and Cancer (MDC)” [22] cohort was used. In this cohort, all men born between 1923 and 1945 and all women born between 1923 and 1950, living in the city of Malmö were invited to participate in a screening examination between 1991 and 1996. A total of 30,446 subjects participated; participation rate was approximately 41%. After exclusion of individuals with history of AF at baseline (*n* = 312), missing anthropometric measures, missing biological or life-style co-variables or missing follow-up (*n* = 4173), a total of 25,961 individuals (9883 men; 16,078 women) were included in the study population. Mean BMI in the excluded group was 26.6 kg/m^2^ and mean age was 59.6 years. The cumulative incidence rate of AF among the individuals excluded from the present study was 17% (761 out of 4485).

The overall mortality in participants was significantly lower compared to the non-participants; however, socioeconomic structure and prevalence of smoking and obesity has been shown to be comparable with the overall population [23].

### Baseline examinations

The examinations were performed by trained nurses at the screening center. The subjects underwent measurement of blood pressure and anthropometric measures, completed a self-administered questionnaire and sampling of peripheral venous blood. Height was measured in a standing position with a fixed stadiometer, calibrated in centimeters (cm). Weight was rounded off to the nearest 0.1 kg using a balance-beam scale, with the subjects wearing light clothing and no shoes. BMI was calculated as weight (kg) divided by the square of the height (m^2^). Waist was measured as the circumference (cm) between the lowest rib and the iliac crest. Hip circumference (cm) was measured as the largest circumference between waist and thighs. WHR was defined as the waist circumference divided by the hip circumference. WHtR was defined as the waist circumference divided by the height.

Bioelectric Impedance Analyzers were used (BIA) for estimating body composition, and BF % was calculated using a mathematical algorithm, according to the procedure provided by the manufacturer (BIA 103, RJL systems, single-frequency analyzer, Detroit, USA).

Data on use of lipid-lowering, antihypertensive and anti-diabetic medications, smoking habits, alcohol consumption, leisure-time physical activity, education level, civil status and immigrant status was obtained through a self-admitted questionnaire.

Blood pressure was measured using a mercury-column sphygmomanometer after 10 min of rest in a supine position. Leukocyte concentration was analyzed consecutively in fresh heparinized blood. Diabetes mellitus was defined as self-reported physician’s diagnosis of diabetes or use of anti-diabetic medications or a diagnosis of diabetes in local or national patient registers. Low leisure-time physical activity was defined as the lowest third of a score calculated from 18 questions on physical activity in four seasons.

Subjects were categorized into smokers (occasional or habitual) and non-smokers (ex-smokers and never-smokers). High alcohol consumption was defined as > 40 g per day for men and > 30 g per day for women. Educational level was divided into three categories: < 9 years (primary education), 9–12 years (some/completed secondary education) and > 12 years (education at college or university level). Civil status was categorized into married and not married. Immigration was classified as Swedish-born and foreign born.

### Ascertainment of AF

All subjects were followed from the baseline examination until first diagnosis of AF, death, emigration or 31st December 2016, whichever came first. Patients were followed by data linkage with the Swedish Hospital Discharge Register (HDR), the Swedish hospital-based out-patient register and the Swedish Cause of Death Register (CDR), which are administered by the Swedish National Board of Health and Welfare. These registers include all residents of Sweden, and there was no missing data at the stage of data linkage. Malmö University Hospital has reported to the HDR since 1969 and outpatient diagnoses have been reported since 2001. The CDR contains diagnoses from death certificates since 1952.

AF was defined as diagnosis code 427.92 (ICD-8, used up to 1986), 427D (ICD-9, used between 1987 and 1996) and I48 (ICD-10, used 1997–2016) [24]. AF was defined as atrial fibrillation or atrial flutter, since these conditions have a close relationship [25].

### Statistical analysis

Cox proportional hazards regression was used to examine the association between anthropometric measurements and incidence of AF. The anthropometric measurements used were BMI, WC, WHR, WHtR, BF %, weight and height, which were divided into quartiles with sex-specific quartile limits.

In a second analysis, BMI and WC were divided into commonly used risk-groups. For BMI, “underweight” was defined as BMI < 18.5 kg/m^2^, “normal weight” as 18.5 ≤ BMI < 25, “overweight” as 25 ≤ BMI < 30, “obese “ as 30 ≤ BMI < 35 and “severe obesity “ as BMI ≥ 35 in both sexes. For WC “Normal” was defined as WC < 94 cm in men and WC < 80 cm in women, “overweight” as 94 ≤ WC < 102 in men and 80 ≤ WC < 88 in women and “obese” as WC ≥ 102 cm in men and WC ≥ 88 cm in women.

The time axis was follow-up time until death, incident AF, emigration from Sweden, or end of follow-up (December 31st, 2016). Hazard ratios (HR) were calculated with 95% confidence intervals (CI). Analyses were performed using three models. The first model adjusted for age. The second model additionally adjusted for and biological risk factors (systolic blood pressure, leukocyte counts, use of antihypertensive and/or lipid-lowering drugs, diabetes mellitus, Apo A1, Apo B, smoking and physical activity). Finally, a third model also included socioeconomic factors (marital status, immigration status, high alcohol consumption and education). Since AF could be secondary to heart failure (HF) or acute myocardial infarction (AMI), we also performed a sensitivity analysis of incident AF in cases without preceding AMI or HF. In this analysis, all individuals were followed until death, incident AF, first diagnosis of heart failure (HF, ICD-10 code I50) or myocardial infarction (AMI ICD-10 code I21-22), date of emigration or December 31^st^, 2016, whichever came first. In this analysis, those with HF or AMI before baseline examination were excluded.

The proportional hazards assumption was assessed visually using Kaplan–Meier graphs, and found to be valid. Interaction between anthropometric measures and age were examined using multiplicative interaction terms.

To evaluate model discrimination for different anthropometric measures, we calculated Harrell’s c-statistics for the anthropometric measures, with risk factor adjustments in the third model [26]. Since BMI is the most widely used measure of overall obesity, we used BMI as reference and present change of C-statistics (ΔC-statistics) when other anthropometric measures are applied. SPSS version 25 (SPSS Inc., Chicago, IL, USA) and STATA (version 12. StataCorp LLC, TX, USA) were used for statistical analyses.

## Results

### Study cohort

The characteristics of the final study population are presented in Table 1. Compared to women, men had higher mean values in all anthropometric measurements except for BF %. Men had higher prevalence of hypertension, smoking, diabetes, being married and had lower education compared to women. 2102 out of 9883 men and 2215 out of 16,078 women developed AF. The incidence was 11.8 (per 1000/person-years) for men and 6.98 (per 1000/person-years) for women. Mean follow up time was 18 ± 6.5 years for men and 19.7 ± 5.3 years for women.

**Table 1:** Baseline characteristics of the study cohort

	Men (n = 9883)	Women (16,078)
Total n:	9883	16,078
Incidence of AF, n (per 1000 p-y)	2102 (11.83)	2215 (6.98)
Age at screening (years)	59 ± 7	57 ± 8
Height (cm)	176.5 ± 6.6	164.7 ± 6.0
Weight (kg)	81.6 ± 12	67.9 ± 11.6
BMI (kg/m2)	26.2 ± 3.4	25.4 ± 4.2
BF %	20.7 ± 5	30.7 ± 5
WHR	0.94 ± 0.06	0.79 ± 0.05
WC (cm)	93.6 ± 12.7	77.7 ± 10.5
WHtR	0.53 ± 0.07	0.48 ± 0.07
Systolic Blood Pressure (mmHg)	144 ± 19	139 ± 20
leukocyte count (10^9/l)	6.37 ± 2.59	6.42 ± 2.32
Follow-up period (years)	18 ± 6.5	19.7 ± 5.3
Apo A1 (mg/dL)	145 ± 24.7	165 ± 27.6
Apo B (mg/dL)	111 ± 25.4	105 ± 26.3
High Alcohol Consumption (> 40/30 g per day for men/women) (%)	7.5	2.4
Current smoking (%)	28.8	27.9
Current use of antihypertensive medicine (%)	17.6	15.30
Use of Lipid Lowering Drugs (%)	3.3	1.80
Immigrated to Sweden (%)	11.9	11.8
Diagnosed diabetes (%)	5.0	3.1
Married (%)	72.7	60.8
Primary school education (%)	45.2	38.9
Secondary school education (%)	19.6	30.5
Higher levels of education (%)	35.2	30.6
History of coronary event (%)	2.9	1.6
History of heart failure (%)	0.4	0.2

Values are means ± standard deviation, unless stated otherwise

*p-y* person years

*AF* Atrial Fibrillation, *BMI* body mass index, *BF %* bodyfat-percentage, *WHR* Waist-Hip Ratio, *WC* Waist Circumference, *WHtR* Waist-Height Ratio, *Apo-A1* apoprotein A1-levels, *Apo B* apoprotein B-levels

Baseline characteristics—divided by gender—are presented for quartiles of BMI and WC in Additional file 1: Tables S1-S2.

### Risk of developing AF in relation to anthropometric measures

High values of BMI, WC, WHR, WHtR, BF %, weight or height were all associated with an increased risk for developing AF in both sexes, in all three models (Table 2a, b).

**Table 2:** ^Incidence of atrial fibrillation in relation to anthropometric measures in (A) men, (B) women^

Quartiles	Q1	Q2	Q3	Q4	Q4 vs. Q1 *p*-value
(A)					
BMI (n)	2460	2474	2471	2478	
AF/1000^a^	9.52	10.7	11.3	16.1	
BMI (median, kg/m^2^)	22.5	24.9	26.9	29.9	
HR-1^b^	1	1.04 (0.91–1.18)	1.11 (0.98–1.26)	1.66 (1.47–1.88)	< 0.001
HR-2^c^	1	1.05 (0.92–1.19)	1.12 (0.98–1.28)	1.61 (1.42–1.83)	< 0.001
HR-3^d^	1	1.05 (0.92–1.19)	1.12 (0.98–1.28)	1.62 (1.42–1.84)	< 0.001
WC (n)	2340	2492	2310	2741	
AF/1000^a^	9.03	10.6	11.8	15.8	
WC (median, cm)	82	90	95	104	
HR-1^b^	1	1.13 (0.99–1.28)	1.24 (1.09–1.42)	1.72 (1.52–1.95)	< 0.001
HR-2^c^	1	1.14 (1.00–1.31)	1.25 (1.09–1.43)	1.68 (1.48–1.92)	< 0.001
HR-3^d^	1	1.14 (1.00–1.30)	1.23 (1.08–1.41)	1.67 (1.46–1.90)	< 0.001
WHR (n)	2457	2457	2482	2487	
AF/1000^a^	11.0	11.4	11.5	13.5	
WHR (median)	0.88	0.92	0.96	1.01	
HR-1^b^	1	1.06 (0.94–1.20)	1.09 (0.96–1.23)	1.39 (1.23–1.57)	< 0.001
HR-2^c^	1	1.05 (0.93–1.19)	1.04 (0.92–1.18)	1.30 (1.15–1.47)	< 0.001
HR-3^d^	1	1.05 (0.93–1.19)	1.04 (0.92–1.18)	1.3 (1.14–1.47)	< 0.001
WHtR (n)	2467	2455	2483	2478	
AF/1000^a^	9.83	10.7	11.6	15.6	
WHtR (median)	0.47	0.51	0.54	0.59	
HR-1^b^	1	1.02 (0.89–1.15)	1.07 (0.94–1.21)	1.45 (1.28–1.64)	< 0.001
HR-2^c^	1	1.00 (0.88–1.14)	1.05 (0.92–1.19)	1.35 (1.19–1.54)	< 0.001
HR-3^d^	1	1.00 (0.88–1.14)	1.05 (0.92–1.19)	1.37 (1.20–1.56)	< 0.001
BF % (n)	1912	2339	3158	2474	
AF/1000^a^	10.9	10.3	12.0	14.0	
BF % (median)	15	18	21	26	
HR-1^b^	1	0.91 (0.79–1.04)	1.07 (0.94–1.21)	1.27 (1.12–1.44)	< 0.001
HR-2^c^	1	0.90 (0.79–1.04)	1.04 (0.92–1.19)	1.22 (1.07–1.39)	< 0.005
HR-3^d^	1	0.90 (0.79–1.04)	1.04 (0.92–1.18)	1.21 (1.06–1.39)	< 0.005
Weight (n)	2162	2741	2477	2503	
AF/1000^a^	8.49	10.7	12.2	15.7	
Weight (median, kg)	68	77	84	95	
HR-1^b^	1	1.23 (1.08–1.42)	1.46 (1.28–1.68)	2.06 (1.80–2.35)	< 0.001
HR-2^c^	1	1.26 (1.10–1.44)	1.49 (1.29–1.71)	2.06 (1.80–2.36)	< 0.001
HR-3^d^	1	1.25 (1.08–1.43)	1.46 (1.27–1.68)	2.02 (1.76–2.32)	< 0.001
Height (n)	2204	2179	2822	2678	
AF/1000^a^	10.4	11.1	12.2	13.1	
Height (median, cm)	168	174	178	184	
HR-1^b^	1	1.16 (1.01–1.33)	1.33 (1.17–1.51)	1.62 (1.43–1.84)	< 0.001
HR-2^c^	1	1.16 (1.02–1.33)	1.38 (1.21–1.56)	1.69 (1.49–1.92)	< 0.001
HR-3^d^	1	1.16 (1.01–1.32)	1.36 (1.2–1.54)	1.66 (1.46–1.89)	< 0.001
^(B)^					
BMI (n)	4016	4023	4008	4031	
AF/1000^a^	4.82	5.87	7.44	10.00	
BMI (median, kg/m^2^)	21.1	23.5	26.0	30.1	
HR-1^b^	1	1.08 (0.95–1.24)	1.22 (1.08–1.40)	1.61 (1.42–1.82)	< 0.001
HR-2^c^	1	1.09 (0.95–1.25)	1.22 (1.07–1.39)	1.51 (1.32–1.72)	< 0.001
HR-3^d^	1	1.09 (0.96–1.25)	1.22 (1.07–1.40)	1.52 (1.33–1.73)	< 0.001
WC (n)	3500	4231	3994	4353	
AF/1000^a^	4.58	5.77	7.04	10.3	
WC (median. cm)	67	72	79	89	
HR-1^b^	1	1.13 (0.98–1.29)	1.29 (1.12–1.48)	1.71 (1.50–1.95)	< 0.001
HR-2^c^	1	1.13 (0.98–1.30)	1.30 (1.13–1.49)	1.62 (1.42–1.86)	< 0.001
HR-3^d^	1	1.13 (0.98–1.30)	1.31 (1.14–1.50)	1.63 (1.422–1.88)	< 0.001
WHR (n)	3991	4034	3999	4054	
AF/1000^a^	6.31	6.32	6.69	8.71	
WHR (median)	0.74	0.77	0.80	0.85	
HR-1^b^	1	1.05 (0.93–1.19)	1.05 (0.93–1.19)	1.33 (1.19–1.50)	< 0.001
HR-2^c^	1	1.05(0.93–1.18)	1.03 (0.91–1.16)	1.24 (1.09–1.40)	0.001
HR-3^d^	1	1.05 (0.93–1.19)	1.03 (0.91–1.12)	1.24 (1.09–1.40)	0.001
WHtR (n)	4006	4032	4018	4022	
AF/1000^a^	4.57	6.06	7.49	10.1	
WHtR (median)	0.41	0.45	0.48	0.55	
HR-1^b^	1	1.11 (0.97–1.27)	1.23 (1.08–1.40)	1.51 (1.33–1.71)	< 0.001
HR-2^c^	1	1.10 (0.96–1.26)	1.21 (1.06–1.38)	1.38 (1.20–1.58)	< 0.001
HR-3^d^	1	1.10 (0.96–1.26)	1.22 (1.06–1.39)	1.39 (1.21–1.59)	< 0.001
BF % (n)	3113	4493	3711	4761	
AF/1000^a^	4.55	5.76	6.88	9.98	
BF % (median)	24	29	32	36	
HR-1^b^	1	1.11 (0.96–1.28)	1.12 (0.966–1.29)	1.53 (1.34–1.75)	< 0.001
HR-2^c^	1	1.14 (0.98–1.32)	1.13 (0.97–1.31)	1.44 (1.25–1.66)	< 0.001
HR-3^d^	1	1.14 (0.98–1.32)	1.13 (0.97–1.31)	1.45 (1.26–1.67)	< 0.001
Weight (n)	3744	3812	4255	4267	
AF/1000^a^	4.92	5.52	7.10	10.13	
Weight (median, kg)	56	63	69	80	
HR-1^b^	1	1.12 (0.97–1.28)	1.41 (1.24–1.6)	2.00 (1.77–2.26)	< 0.001
HR-2^c^	1	1.13 (0.98–1.30)	1.44 (1.26–1.64)	1.93 (1.70–2.20)	< 0.001
HR-3^d^	1	1.13 (0.98–1.30)	1.44 (1.27–1.65)	1.93 (1.70–2.20)	< 0.001
Height (n)	3920	3992	3915	4251	
AF/1000^a^	6.72	6.74	7.02	7.41	
Height (median, cm)	157	162	165	170	
HR-1^b^	1	1.16 (1.03–1.32)	1.37 (1.21–1.55)	1.82 (1.62–2.06)	< 0.001
HR-2^c^	1	1.19 (1.19–1.06)	1.40 (1.24–1.58)	1.90 (1.68–2.14)	< 0.001
HR-3^d^	1	1.20 (1.06–1.36)	1.42 (1.26–1.61)	1.93 (1.71–2.18)	< 0.001

^a^Measured as number of AF per 1000-person years

^b^Cox-regression Hazard Ratio (HR) adjusted for age (95% CI)

^c^HR-2 adjusted for age, use of antihypertensive medication, lipid-lowering medication, systolic blood pressure, smoking, low physical activity, Apo-A and Apo-B blood levels and diabetes (95% CI)

^d^HR-3 adjusted for HR-2 plus alcohol consumption, low education, marital status and immigrant status (95% CI)

*AF* Atrial Fibrillation, *BMI* body mass index, *WC* waist circumference, *WHR* waist-hip ratio, *WHtR* waist-height ratio, *BF %* body-fat percentage, *CI* confidence interval

The incidence of AF, measured as incidence per thousand person-years increased from the first quartile to the fourth quartile for all anthropometric measures in both men and women (Table 2a, b). Kaplan–Meier graphs are presented in Additional file 1: Figure S3A-G and S4A-G.

**Fig. 1:**
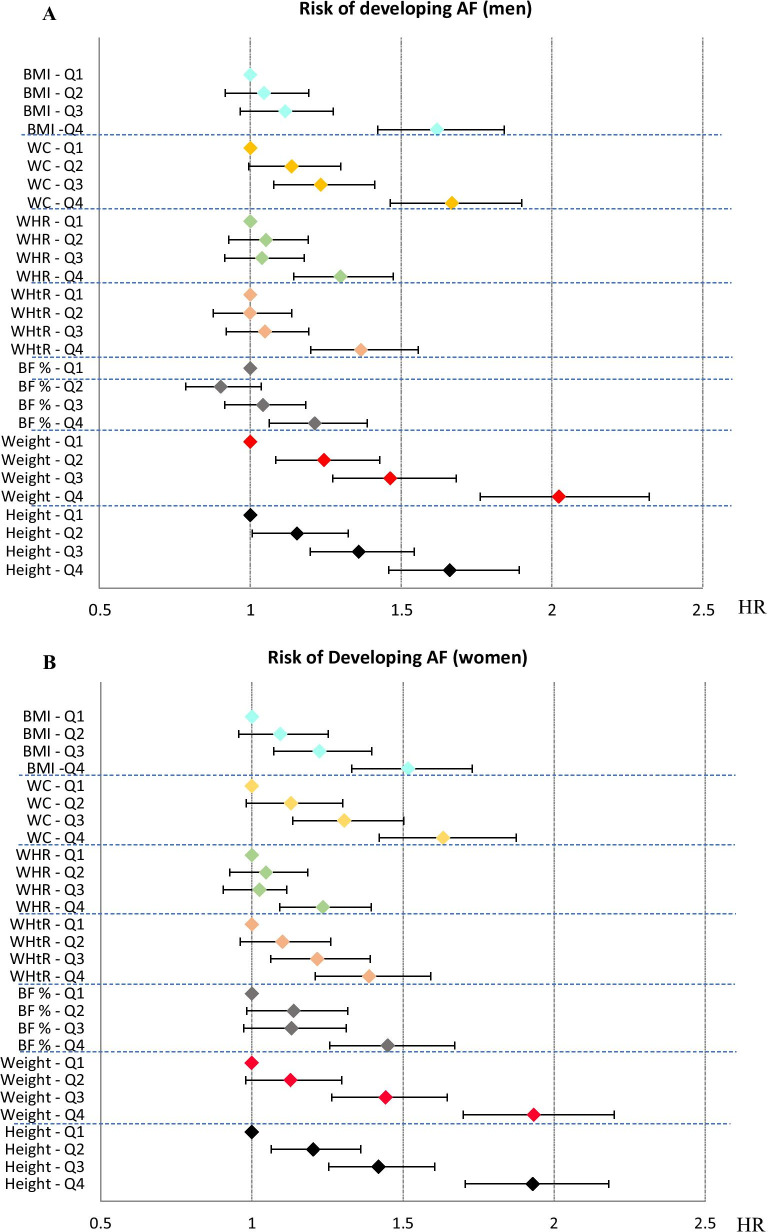
**a** Graphic representation of Hazard Ratios (HRs) for developing atrial fibrillation (AF) for each anthropometric measure in men. HRs are adjusted for age, use of antihypertensive medication, lipid-lowering medication, systolic blood pressure, smoking, low physical activity, Apo-A and Apo-B blood levels, diabetes, alcohol consumption, low education, marital status and immigrant status. HRs are represented with a colored rhomb. Brackets represent 95% confidence intervals. Hazard Ratios for Quartiles of Anthropometric Measures in Men. *BMI* body mass index, *WC* waist circumference, *WHR* waist hip ratio, *WHtR* waist height ratio, *BF %* body fat percentage, *Q* quartile, *HR* hazards ratio. **b** Graphic representation of Hazard Ratios (HRs) for developing atrial fibrillation (AF) for each anthropometric measure in women. HRs are adjusted for age, use of antihypertensive medication, lipid-lowering medication, systolic blood pressure, smoking, low physical activity, Apo-A and Apo-B blood levels, diabetes, alcohol consumption, low education, marital status and immigrant status. HRs are represented with a colored rhomb. Brackets represent 95% confidence intervals. Hazard Ratios for Quartiles of Anthropometric Measures in Women. *BMI* body mass index, *WC* waist circumference, *WHR* waist hip ratio, *WHtR* waist height ratio, *BF %* body fat percentage, *Q* quartile, *HR* hazards ratio

The HRs of incident AF in men (fourth vs. first quartile) were 1.62 for BMI, 1.67 for WC, 1.30 for WHR, 1.37 for WHtR, 1.21 for BF %, 2.02 for weight and 1.67 for height, with adjustments in the third model. For women, the HRs of incident AF were 1.52 for BMI, 1.63 for WC, 1.24 for WHR, 1.39 for WHtR, 1.45 for BF %, 1.93 for weight and 1.92 for height. All HRs were significant, with *p*-values (first vs. fourth quartile) ≤ 0.001. *P*-values for trends across quartiles were also statistically significant (*p* ≤ 0.002). The results are presented graphically in Fig. 1a, b.

There were no significant interactions between anthropometric measures and age in men (*p* > 0.38). However, in women there were significant interactions between age and weight (*p* for interaction < 0.01), BMI (*p* = 0.03) and BF % (*p* < 0.01), respectively, indicating stronger effects of obesity in younger women (below median age 57 years) compared to older women (> 57 years).

Incidence of AF in relation to commonly used cut-offs for BMI and WC are shown in Additional file 1: Figure S5A-B.

C-statistics for weight were significantly higher than for BMI in men and women. C-statistics for WHR, WHtR and BF % was significantly lower than for BMI in men and women (Table 3).

**Table 3:** Comparison of HRs for different anthropometric measures

Men	HR	Δ C-statistics	*p*-value	Women	HR	Δ C-statistics	*p*-value
BMI	1.18 (1.13–1.23)			BMI	1.15 (1.11–1.2)		
Weight	1.26 (1.21–1.32)	0.005 (0.002–0.008)	< 0.01	Weight	1.26 (1.21–1.31)	0.005 (0.004–0.007)	< 0.01
BF %	1.08 (1.04–1.13)	− 0.004 (− 0.006 to − 0.001)	0.01	BF %	1.13 (1.08–1.18)	− 0.003 (− 0.004 to − 0.001)	0.01
WHR	1.08 (1.04–1.13)	− 0.004 (− 0.007 to − 0.002)	< 0.01	WHR	1.07 (1.02–1.11)	− 0.003 (− 0.004 to − 0.001)	0.01
WC	1.18 (1.14–1.23)	0.001 (− 0.001 to 0.003)	0.55	WC	1.18 (1.13–1.23)	0.001 (− 0.001 to 0.002)	0.34
WHtR	1.11 (1.06–1.16)	− 0.003 (− 0.005 to − 0.001)	< 0.01	WHtR	1.12 (1.07–1.17)	− 0.002 (− 0.003 to − 0.001)	< 0.01
Height	1.19 (1.14–1.24)	0.001 (− 0.004 to 0.005)	0.76	Height	1.24 (1.19–1.29)	0.004 (0.001–0.008)	0.02

All ΔC-statistics and p values refer to difference between the anthropometric measure and BMI. C-statistics for BMI was xx for men and yy for women

Cox regression Hazard Ratio (trend) of BMI, Weight, BF %, WHR, WC, WHtR, Height (adjusted for age, use of antihypertensive medication, lipid-lowering medication, systolic blood pressure, smoking, low physical activity, Apo-A and Apo-B blood levels, diabetes, alcohol consumption, low education, marital status and immigrant status) in men (orange) and women (blue)

*BMI* body mass index, *WC* waist circumference, *WHR* waist-hip ratio, *WHtR* waist-height ratio, *BF %* body-fat percentage

A sensitivity analysis was performed wherein individuals with incident AMI or HF were followed until the date of AMI or HF diagnosis, and censored after that. A total of 3099 cases of incident AF (1439 men and 1660 women) remained in this analysis. The adjusted HR for incident AF in men, without preceding AMI or HF, were (fourth vs. first quartile) 1.76 for BMI, 1.74 for WC, 1.34 for WHR, 1.44 for WHtR, 1.20 for BF %, 2.02 for weight and 1.53 for height. For women, the corresponding HRs were 1.47 for BMI, 1.58 for WC, 1.30 for WHR, 1.35 for WHtR, 1.46 for BF %, 1.91 for weight and 1.94 for height (Additional file 1: Table S6A and B).

To summarize the results, all anthropometric measures were positively associated with an increased risk for developing AF. The effect was higher for BMI and weight compared to WHR and BF %, and weight showed the best model discrimination of all anthropometric measures. The incidence of AF was highest in the fourth quartile of each anthropometric measure in both men and women.

## Discussion

Obesity has previously been associated with higher risk of developing AF [4, 5, 7–9, 12, 20], and high BMI, height, weight, WC and BF % have all been associated with an increased risk of AF [9, 20, 27–29]. This study confirms the association between obesity and the risk of developing AF. BMI, height, weight, WC, WHR, WHtR and BF % were all risk factors for AF, supporting the results from a similar Danish cohort study [20]. However, HRs comparing the 4th versus 1st quartiles of anthropometric measures indicated that overall weight (e.g. BMI and weight) was more predictive than measures of abdominal obesity (e.g. WHR and WHtR).

Our data suggests that weight is a slightly better predictor of AF than BMI, which is better than BF %, WHR and WHtR in both men and women. Weight and height are correlated, and both have independently been associated with increased risk of AF [29]. This could explain why weight was a slightly better predictor than BMI.

Although the underlying biological mechanism is uncertain, it has been shown that obesity is associated with increased heart volume—especially left atrial volume—which has been proved a precursor for AF [5]. Additionally, tall non-obese individuals have bigger hearts and increased risk of AF and some studies show that height itself could be a risk factor for AF [29]. It is thought that increased left atrial volume is correlated with increased number of cardiomyocytes in the pulmonary sleeves, triggering AF. As previously mentioned, obesity is also associated with increased pericardial tissue, which is a risk for structural changes within the heart that alter the cardiac electrical circuits, causing AF [30]. In addition, obesity–related hypertension as well as various obesity-related hormones, such as leptin, adiponectin and tumor necrosis factor α [31] could also be common links between obesity, cardiac remodeling and increased risk of AF [32].

Due to the increasing prevalence of obesity [10, 33], the occurrence, morbidity and mortality of AF is expected to increase globally. Hence, it is important to understand which anthropometric measures that can be used to evaluate the individual risk for AF. For some diseases, e. g. diabetes, it has been shown that abdominal adiposity is a strong risk factor, and that fat distribution is of great importance [4, 34]. In contrast, the results of this study suggest that overall obesity is more important than abdominal obesity for the risk of developing AF.

Obesity is also a well-documented risk factor for hypertension and diabetes [34]. Although we adjusted for hypertension and diabetes at the baseline examination, it was not possible to adjust for these risk factors during the follow-up period. It is therefore still possible that incidence of hypertension and diabetes could contribute to the increased risk of AF in obese individuals. Further, obesity is also a risk factor for other conditions associated with AF, such as obstructive sleep apnea, heart failure and myocardial infarction, all of which are risk factors for AF. However, our analysis were essentially unchanged when we analyzed AF without prior myocardial infarction or heart failure in a sensitivity analysis. These factors could also be regarded as parts of the causal chain between obesity and AF. Additionally, some studies suggest that weight reduction can decrease the cardiac volume and the burden of symptoms, namely the frequency and duration of AF episodes in patients with AF [7], suggesting that weight control can be of value with manifest AF as well.

This study has several strengths: it is a population-based study, with a large number of participants that have been followed for more than 16–18 years. The case validity for AF-diagnosis in the MDC-cohort has been shown to be high [24]. The patients have been followed by linkage with the Swedish patient registers. However, AF is sometimes subclinical and undetected, and we cannot rule out that some cases have been missed, but since most individuals in this age-group visit a doctor at some time-point it is likely that most cases will be identified by the registers. The relatively high cumulative incidence of AF in the study (16.6%) supports this view.

Participants with incident AF were on average less healthy than those without AF and residual confounding is always a potential cause of bias in observational studies. Although several baseline characteristics have been adjusted for there are still potential confounders, such as thyroid disease or pulmonary diseases, that have not been adjusted for. This is a limitation to this study.

Another possible limitation in this study is that obese individuals are more prone to seek medical care, due to co-morbid conditions, than non-obese individuals. This might result in more cases of diagnosed AF in the obese population than in the non-obese population. However, our results are in accordance to those from other studies on the subject [9, 20]. Another limitation is that no ECG were taken at the baseline examination and that some individuals could have had subclinical and undetected AF. However, prevalence of AF is low in this age-group (mean 58 years) and it can be assumed that the number of undetected cases of AF is small.

The group of incident AF consists of several subgroups, such as paroxysmal AF, persistent AF and atrial flutter, which have not been analyzed separately. This must be taken into consideration, since these subtypes theoretically could have different relationships with anthropometric measures.

## Conclusion

The results of this study indicate that an increase in weight, height, BMI, WC, WHR, WHtR and BF % all increase the risk of AF in both sexes, confirming previous results from similar studies. In contrast to several other cardiometabolic diseases, where abdominal obesity is associated with a higher risk, weight and general obesity seems to be more important than abdominal obesity as a risk factor for developing AF.

## Supplementary Information

**Additional file 1**. Supplementary data.

## Data Availability

The data that support the findings of this study are available from “The Malmö Cohorts” at Lund’s University (https://www.malmo-kohorter.lu.se/malmo-cohorts) upon reasonable request and with permission of the steering committee.
