# Supplementary material for: Anthropometric measures and the risk of developing atrial fibrillation: a Swedish Cohort Study
Source: BMC Cardiovasc Disord. 2021 Dec 18;21:602. doi: 10.1186/s12872-021-02415-6 (PMC8684176; doi:10.1186/s12872-021-02415-6)
Supplement: Supplementary file 1 — Additional file 1. Supplementary data. [file 12872_2021_2415_MOESM1_ESM.docx]

**Supplementary Data**

# **Table S1A – S1B (BMI Quartile Characteristics)**

| **Men BMI Quartile** | **Q1** | **Q2** | **Q3** | **Q4** |
| --- | --- | --- | --- | --- |
| Total (n) | 2460 | 2474 | 2471 | 2478 |
| Developed AF (n) | 426 | 489 | 506 | 681 |
| Age (yrs) | 58 ± 7 | 59 ± 7 | 59 ± 7 | 59 ± 7 |
| BMI (kg/m^2) | 22.14 ± 1.41 | 24.92 ± 0.59 | 26.96 ± 0.64 | 30.65 ± 2.45 |
| WC (cm) | 83.8 ± 16.9 | 90.1 ± 5.0 | 95.3 ± 5.2 | 104.9 ± 8.1 |
| WHtR | .47 ± 0.09 | .51 ± 0.03 | .54 ± 0.03 | .60 ± 0.04 |
| WHR | .90 ± 0.05 | .93 ± 0.04 | .95 ± 0.05 | .99 ± 0.05 |
| BF % | 17.4 ± 4.0 | 19.5 ± 3.8 | 21.4 ± 4.0 | 24.4 ± 5.0 |
| Weight (kg) | 69.6 ± 7.0 | 77.9 ± 5.9 | 83.8 ± 6.4 | 95.2 ± 10.4 |
| Height (cm) | 177.15 ± 6.72 | 176.66 ± 6.44 | 176.17 ± 6.56 | 176.09 ± 6.55 |
| LKC count (10^9/l) | 6.38 ± 2.42 | 6.26 ± 1.74 | 6.30 ± 1.71 | 6.52 ± 3.88 |
| Follow-up period (years) | 18.2 ± 6.5 | 18.5 ± 6.3 | 18.1 ± 6.4 | 17.1 ± 6.8 |
| Apo A1 (mg/dL) | 152.0 ± 26.8 | 146.7 ± 25.0 | 143.3 ± 22.7 | 139.8 ± 22.3 |
| Apo B (mg/dL) | 103.5 ± 24.8 | 110.7 ± 25.1 | 113.3 ± 24.3 | 115.1 ± 26.0 |
| High Alcohol Consumption (> 40/30 gram per day for men/women) (%) | 6.6 | 7.2 | 7.1 | 9.0 |
| Current smoking (%) | 39 | 29 | 25 | 23 |
| Use of antihypertensive drugs (%) | 9.9 | 14.6 | 19.1 | 27.0 |
| Use of lipid lowering drugs (%) | 2.8 | 3.1 | 3.7 | 3.8 |
| Systolic Blood Pressure (mmHg) | 139 ± 19 | 143 ± 19 | 145 ± 19 | 148 ± 18 |
| Diagnosed Diabetes (%) | 3.3 | 4.0 | 4.6 | 8.2 |
| Immigrated to Sweden (%) | 9.1 | 10.7 | 12.5 | 15.5 |
| Married (%) | 68.7 | 74.3 | 76.1 | 71.6 |
| History of Coronary Event (%) | 2.8 | 2.6 | 3.1 | 2.9 |
| History of Heart Failure (%) | 0.3 | 0.2 | 0.7 | 0.4 |
| **Supplementary Table 4A – BMI Quartile Specific Characteristics for men**  AF = Atrial Fibrillation; BMI = body mass index; BF% = bodyfat-percentage; WHR = Waist-Hip Ratio; WC = Waist Circumference; WHtR = Waist-Height Ratio; Apo-A1 = apoprotein A1-levels; Apo B = apoprotein B-levels Values are means ± standard deviation, unless stated otherwise | | | | |

# **Table S1A – S1B (BMI Quartile Characteristics)**

| **Women BMI Quartile** | **Q1** | **Q2** | **Q3** | **Q4** |
| --- | --- | --- | --- | --- |
| Total (n) | 4016 | 4023 | 4008 | 4031 |
| Developed AF (n) | 391 | 475 | 589 | 760 |
| Age (yrs) | 56 ± 8 | 57 ± 8 | 58 ± 8 | 59 ± 8 |
| BMI (kg/m^2) | 20.80 ± 1.30 | 23.55 ± 0.63 | 26.02 ± 0.84 | 31.08 ± 3.28 |
| WC (cm) | 68.1 ± 4.6 | 73.6 ± 4.9 | 78.8 ± 5.5 | 90.3 ± 9.7 |
| WHtR | .41 ± 0.03 | .45 ± 0.03 | .48 ± 0.03 | .56 ± 0.06 |
| WHR | .77 ± 0.4 | .78 ± 0.05 | .80 ± 0.05 | .83 ± 0.06 |
| BF % | 25.3 ± 3.6 | 29.3 ± 2.8 | 32.1 ± 2.7 | 36.1 ± 3.0 |
| Weight (kg) | 56.6 ± 5.5 | 63.5 ± 4.8 | 69.5 ± 5.4 | 82.0 ± 10.2 |
| Height (cm) | 164.79 ± 5.98 | 164.09 ± 5.93 | 163.36 ± 5.96 | 162.41 ± 6.04 |
| LKC count (10^9/l) | 6.27 ± 2.00 | 6.31 ± 2.43 | 6.41 ± 2.08 | 6.68 ± 2.67 |
| Follow-up period (years) | 20.2 ± 5.1 | 20.1 ± 5.1 | 19.8 ± 5.3 | 18.9 ± 5.8 |
| Apo A1 (mg/dL) | 170.5 ± 28.9 | 167.0 ± 27.5 | 163.2 ± 26.9 | 157.3 ± 25.3 |
| Apo B (mg/dL) | 96.2 ± 24.0 | 102.2 ± 25.0 | 107.6 ± 26.5 | 113.1 ± 26.6 |
| High Alcohol Consumption (> 40/30 gram per day for men/women) (%) | 2.6 | 2.8 | 2.4 | 1.9 |
| Current smoking (%) | 36.4 | 28.7 | 24.7 | 21.9 |
| Use of antihypertensive drugs (%) | 8.7 | 11.4 | 15.6 | 25.5 |
| Use of lipid lowering drugs (%) | 0.7 | 1.3 | 1.9 | 3.1 |
| Systolic Blood Pressure (mmHg) | 134 ± 19 | 137 ± 20 | 141 ± 20 | 146 ± 20 |
| Diagnosed Diabetes (%) | 1.5 | 1.6 | 2.8 | 6.3 |
| Immigrated to Sweden (%) | 9.2 | 10.2 | 12.2 | 15.6 |
| Married (%) | 57.7 | 61.5 | 62.5 | 61.6 |
| History of Coronary Event (%) | 1.8 | 1.6 | 1.6 | 1.4 |
| History of Heart Failure (%) | 0.2 | 0.2 | 0.1 | 0.3 |
| **Supplementary Table 4B – BMI Quartile Specific Characteristics for women**  AF = Atrial Fibrillation; BMI = body mass index; BF% = bodyfat-percentage; WHR = Waist-Hip Ratio; WC = Waist Circumference; WHtR = Waist-Height Ratio; Apo-A1 = apoprotein A1-levels; Apo B = apoprotein B-levels Values are means ± standard deviation, unless stated otherwise | | | | |

# **Table S2A – S2B (WC Quartile Characteristics)**

| **Men WC Quartile** | **Q1** | **Q2** | **Q3** | **Q4** |
| --- | --- | --- | --- | --- |
| Total (n) | 2340 | 2492 | 2310 | 2741 |
| Developed AF (n) | 394 | 492 | 494 | 722 |
| Age (yrs) | 58 ± 7 | 59 ± 7 | 59 ± 7 | 60 ± 7 |
| BMI (kg/m^2) | 22.65 ± 1.96 | 24.99 ± 1.63 | 26.58 ± 1.71 | 29.92 ± 2.89 |
| WC (cm) | 81.4 ± 4.1 | 89.6 ± 1.7 | 95.4 ± 1.70 | 106.1 ± 16.1 |
| WHtR | .47 ± 0.03 | .51 ± 0.02 | .54 ± 0.02 | .60 ± 0.09 |
| WHR | .88 ± 0.04 | .93 ± 0.03 | .95 ± 0.03 | .99 ± 0.05 |
| BF % | 16.7 ± 3.6 | 19.4 ± 3.5 | 21.3 ± 3.8 | 24.6 ± 4.8 |
| Weight (kg) | 69.5 ± 7.0 | 77.4 ± 6.0 | 83.1 ± 6.2 | 94.5 ± 10.3 |
| Height (cm) | 175.15 ± 6.42 | 176.10 ± 6.54 | 176.91 ± 6.43 | 177.73 ± 6.64 |
| LKC count (10^9/l) | 6.27 ± 2.43 | 6.25 ± 1.66 | 6.31 ± 1.71 | 6.60 ± 3.76 |
| Follow-up period (years) | 18.6 ± 6.2 | 18.6 ± 6.3 | 18.2 ± 6.4 | 16.7 ± 6.9 |
| Apo A1 (mg/dL) | 152.7 ± 26.4 | 146.0 ± 24.7 | 143.8 ± 23.7 | 140.0 ± 22.3 |
| Apo B (mg/dL) | 103.1 ± 24.9 | 110.0 ± 24.7 | 113.2 ± 24.0 | 115.5 ± 26.3 |
| High Alcohol Consumption (> 40/30 gram per day for men/women) (%) | 5.9 | 6.5 | 7.6 | 9.6 |
| Current smoking (%) | 33.7 | 29.9 | 26.4 | 25.5 |
| Use of antihypertensive drugs (%) | 2.4 | 3.7 | 3.6 | 3.7 |
| Use of lipid lowering drugs (%) | 140 ± 20 | 142 ± 19 | 145 ± 19 | 148 ± 19 |
| Systolic Blood Pressure (mmHg) | 9.9 | 14.0 | 18.5 | 3.7 |
| Diagnosed Diabetes (%) | 3.3 | 3.0 | 4.7 | 8.5 |
| Immigrated to Sweden (%) | 11.1 | 11.3 | 11.5 | 13.6 |
| Married (%) | 70.6 | 73.6 | 75.7 | 71.1 |
| History of Coronary Event (%) | 3.5 | 2.1 | 3.1 | 2.7 |
| History of Heart Failure (%) | 0.3 | 0.4 | 0.5 | 0.3 |
| **Supplementary Table 2A – WC Quartile Specific Characteristics for men**  AF = Atrial Fibrillation; BMI = body mass index; BF% = bodyfat-percentage; WHR = Waist-Hip Ratio; WC = Waist Circumference; WHtR = Waist-Height Ratio; Apo-A1 = apoprotein A1-levels; Apo B = apoprotein B-levels Values are means ± standard deviation, unless stated otherwise | | | | |

# **Table S2A – S2B (WC Quartile Characteristics)**

| **Women WC Quartile** | **Q1** | **Q2** | **Q3** | **Q4** |
| --- | --- | --- | --- | --- |
| Total (n) | 3500 | 4231 | 3994 | 4353 |
| Developed AF (n) | 327 | 493 | 555 | 840 |
| Age (yrs) | 55 ± 7 | 57 ± 8 | 58 ± 8 | 59 ± 8 |
| BMI (kg/m^2) | 21.29 ± 1.92 | 23.54 ± 1.90 | 25.69 ± 2.19 | 30.12 ± 3.91 |
| WC (cm) | 65.9 ± 2.7 | 72.5 ± 1.70 | 78.7 ± 2.0 | 91.4 ± 8.1 |
| WHtR | .40 ± 0.02 | .44 ± 0.02 | .48 ± 0.02 | .56 ± 0.05 |
| WHR | .75 ± 0.04 | .77 ± 0.04 | .80 ± 0.04 | .84 ± 0.05 |
| BF % | 25.5 ± 3.9 | 29.1 ± 3.3 | 31.8 ± 3.1 | 35.5 ± 3.3 |
| Weight (kg) | 56.5 ± 5.8 | 63.2 ± 5.5 | 69.1 ± 6.20 | 80.6 ± 10.8 |
| Height (cm) | 162.92 ± 5.69 | 163.89 ± 6.05 | 164.06 ± 6.20 | 163.66 ± 6.12 |
| LKC count (10^9/l) | 6.15 ± 1.98 | 6.30 ± 2.46 | 6.43 ± 2.01 | 6.75 ± 2.63 |
| Follow-up period (years) | 20.4 ± 4.8 | 20.2 ± 5.0 | 19.7 ± 5.4 | 18.7 ± 5.9 |
| Apo A1 (mg/dL) | 172.3 ± 28.7 | 167.7 ± 27.7 | 163.0 ± 26.4 | 156.5 ± 25.4 |
| Apo B (mg/dL) | 94.8 ± 23.6 | 100.6 ± 24.2 | 107.4 ± 26.0 | 114.5 ± 26.9 |
| High Alcohol Consumption (> 40/30 gram per day for men/women) (%) | 2.1 | 2.6 | 2.6 | 2.3 |
| Current smoking (%) | 32.1 | 28.6 | 27.7 | 24.2 |
| Use of antihypertensive drugs (%) | 0.6 | 1.0 | 2.1 | 3.2 |
| Use of lipid lowering drugs (%) | 134 ± 19 | 137 ± 20 | 139 ± 20 | 146 ± 20 |
| Systolic Blood Pressure (mmHg) | 7.8 | 11.7 | 14.8 | 25.3 |
| Diagnosed Diabetes (%) | 1.3 | 1.6 | 2.1 | 6.7 |
| Immigrated to Sweden (%) | 9.0 | 11.2 | 12.0 | 14.4 |
| Married (%) | 59.1 | 61.4 | 62.1 | 60.4 |
| History of Coronary Event (%) | 1.7 | 2.0 | 1.3 | 1.5 |
| History of Heart Failure (%) | 0.3 | 0.2 | 0.3 | 0.2 |
| **Supplementary Table 2B – WC Quartile Specific Characteristics for women**  AF = Atrial Fibrillation; BMI = body mass index; BF% = bodyfat-percentage; WHR = Waist-Hip Ratio; WC = Waist Circumference; WHtR = Waist-Height Ratio; Apo-A1 = apoprotein A1-levels; Apo B = apoprotein B-levels Values are means ± standard deviation, unless stated otherwise | | | | |

# **Figure S3A – S3B (Survival Functions, men)**

**
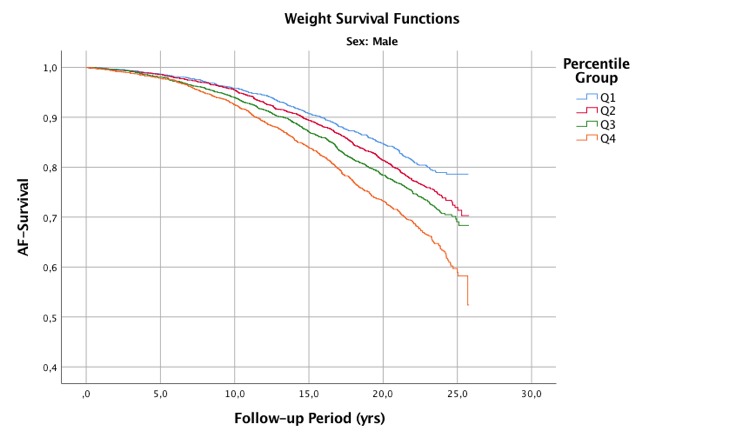

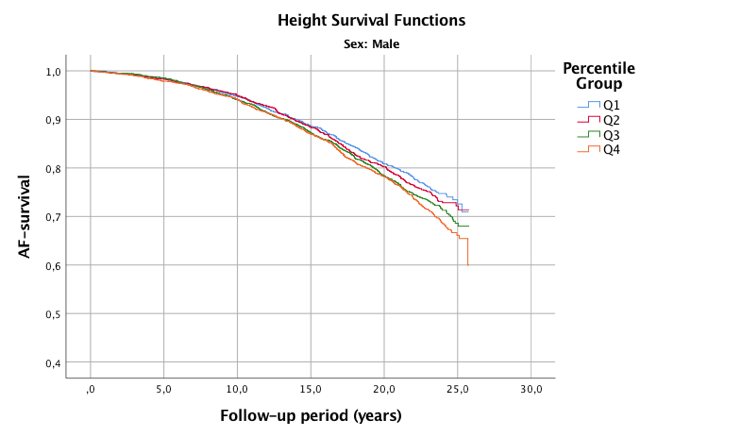
**

**S3B**

**S3A**

# **
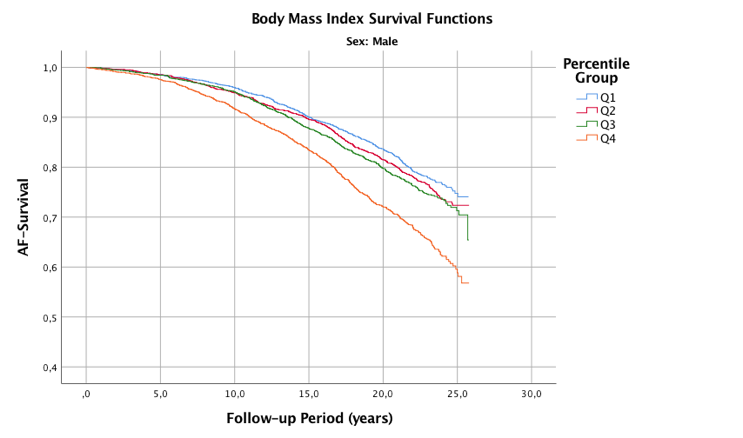
**

**S3C**

# **Figure S3C – S3D (Survival Functions, men)**


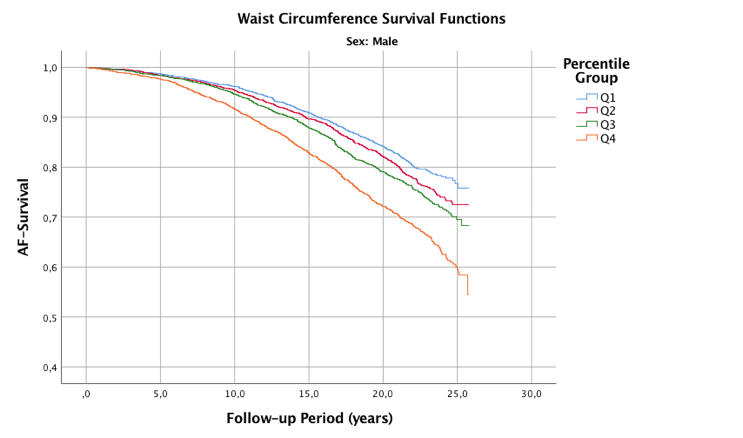
**Figure S3E – S3F (Survival Functions, men)**

**S3D**

**
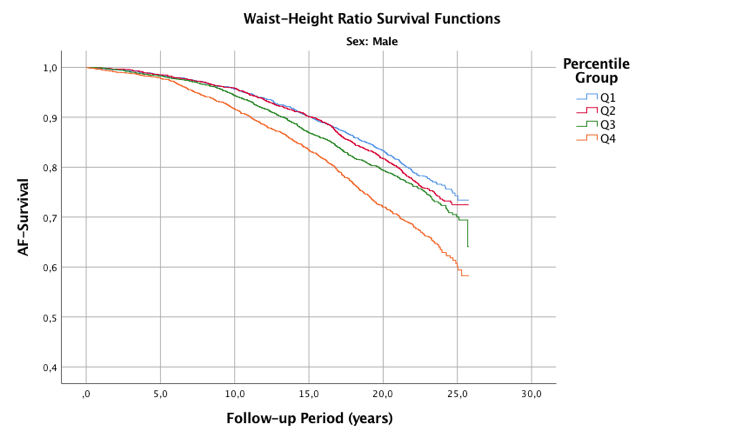

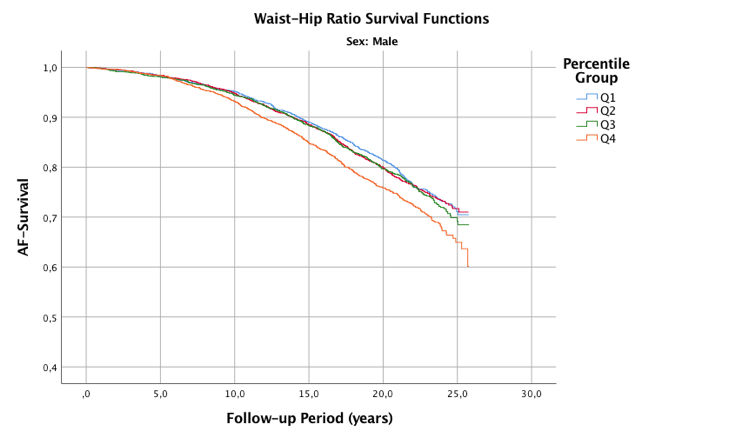
**

**S3F**

**S3E**

# **Figure S3G (Survival Functions, men)**


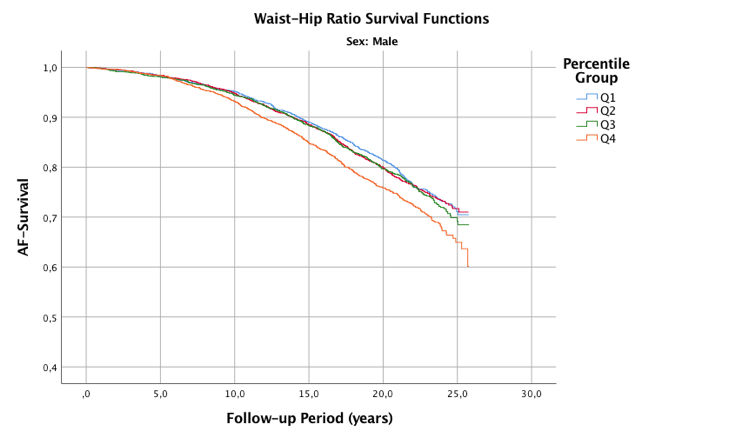

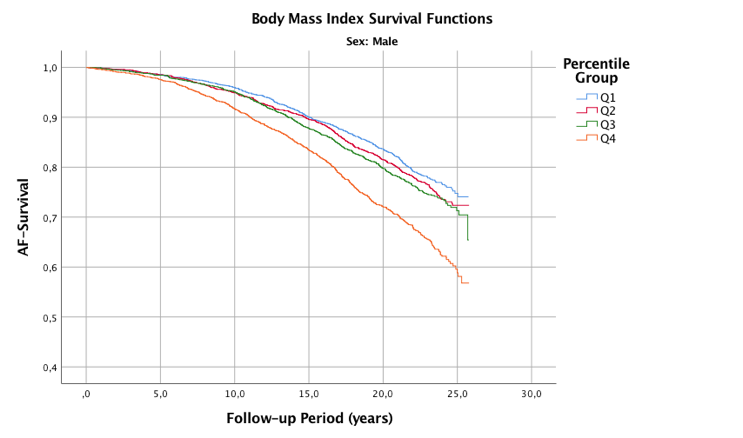


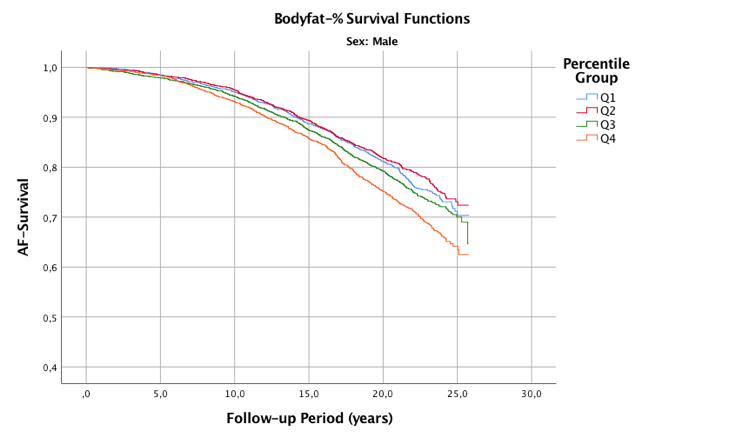


**Figure S3A – S3G**  quartile specific (Q1-Q4) Atrial Fibrillation-survival rates for men for each anthropometric measure (2A Weight, 2B Height, 2C Body Mass Index (BMI), 2D Waist Circumference (WC), 2E Waist-Hip-Ratio (WHR), 2F Waist-Height Ratio (WHtR), 2G Bodyfat-% (BF%). The y-axis describes the survival (1 = 100 %) and the x-axis the follow-up period measured in years.

Survival functions are not adjusted for any co-variables.

**S3G**

# **
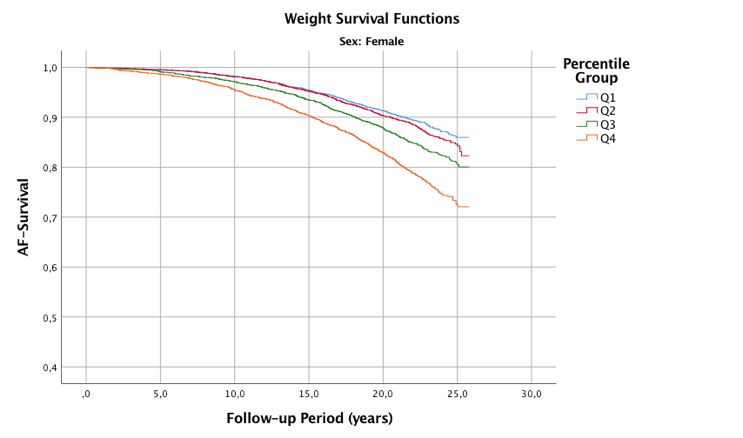

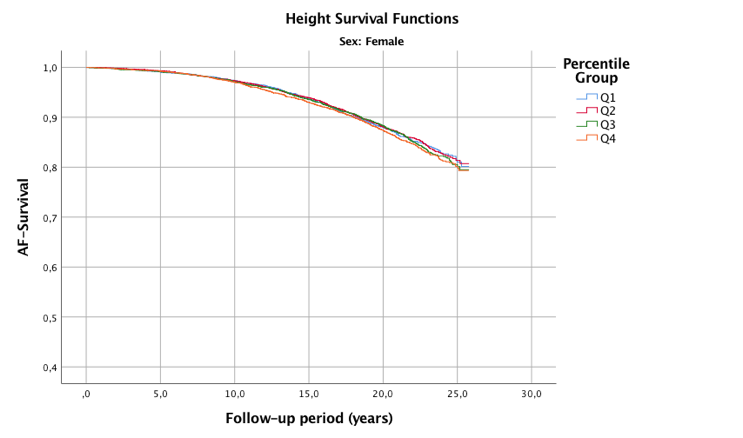
Figure S4A – S4G (Survival Functions, women)**

**S4B**

**S4A**

# **Figure S4A – S4G (Survival Functions, women)**

**
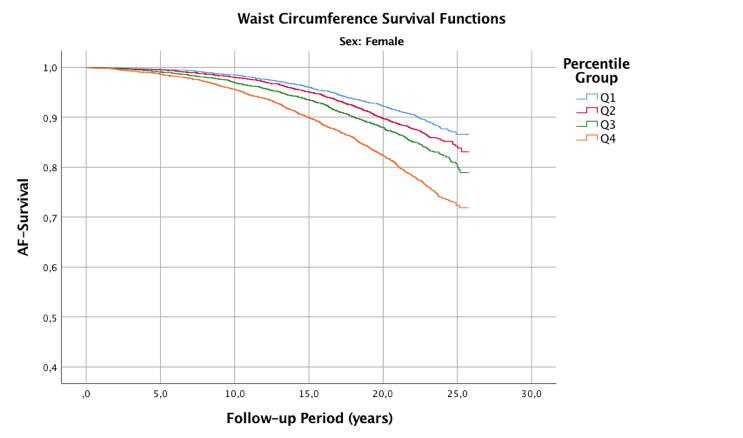

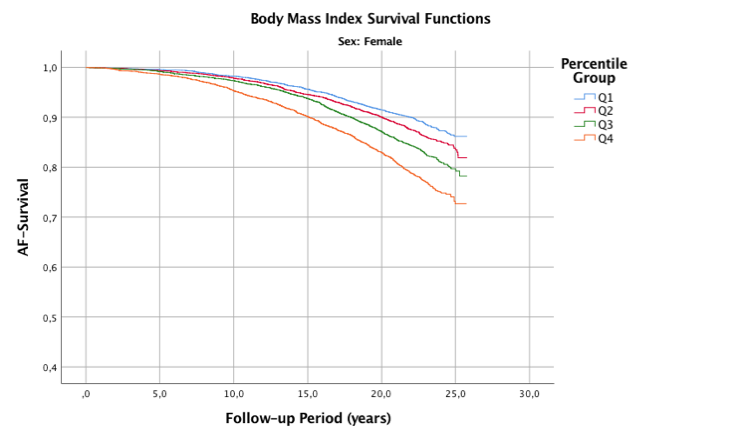
**

**S4D**

**S4C**

# **Figure S4A – S4G (Survival Functions, women)**

**
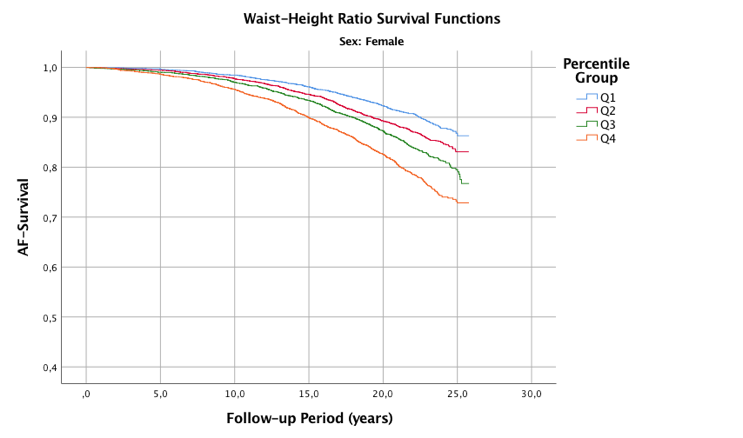

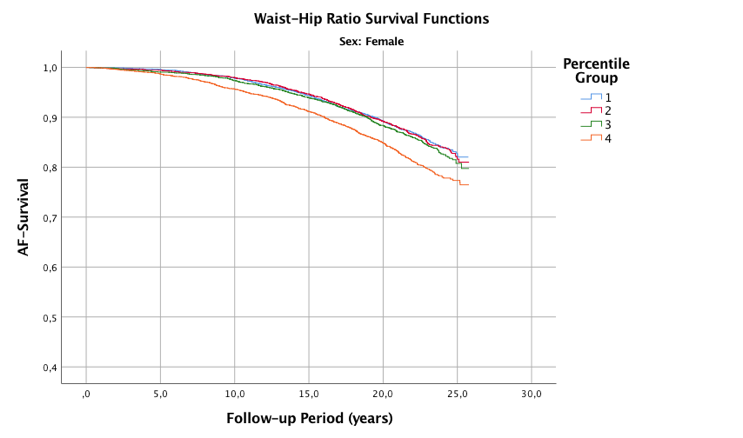
**

**S4F**

**S4E**

# **Figure S4A – S4G (Survival Functions, women)**


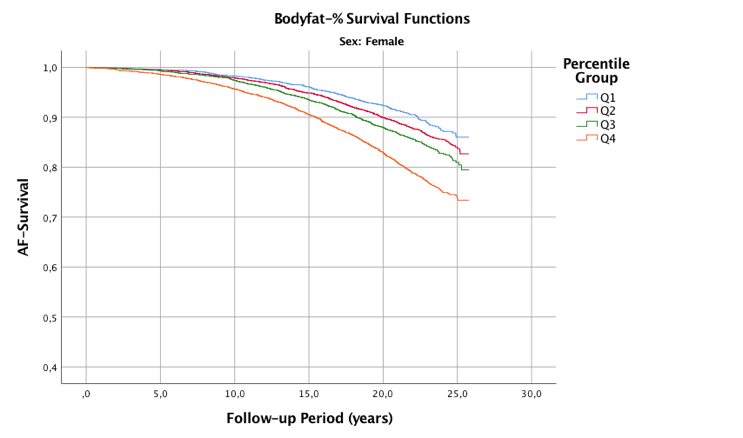


**S4G**

**Figure S4A-S4G**  quartile specific (Q1-Q4) Atrial Fibrillation-survival rates for women for each anthropometric measure (3A Weight, 3B Height, 3C Body Mass Index (BMI), 3D Waist Circumference (WC), 3E Waist-Hip-Ratio (WHR), 3F Waist-Height Ratio (WHtR), 3G Bodyfat-% (BF%). The y-axis describes the survival (1 = 100 %) and the x-axis the follow-up period measured in years.

Survival functions are not adjusted for any co-variables.

# **Figure S5A – S5B – Hazard Ratios in Men (A) and Women (B)**

S5A

HR

S5B

HR

BMI = BMI = body mass index; WC = waist circumference; WHR = waist hip ratio; WHtR = waist height ratio; BF % = body fat percentage; Q = quartile; HR = hazards ratio

**Figure S5A – S5B Hazard Ratios in Men (1A) and Women (1B)**

Figure S1A and S1B show incidence of AF in relation to commonly used cut-offs for BMI and WC. The HRs of incident AF in individuals with 30 ≤ BMI < 35 compared to normal BMI (18.5 ≤ BMI < 25) was 1.65 for men and 1.58 for women, in the third model. In individuals with BMI ≥ 35, the HRs was 2.72 in men and 1.66 in women. For WC ≥102 in men, compared to WC < 94, the HR was 1.65. For WC > 88 in women, compared to WC < 80, the HR was 1.62.

**Table S6A – Hazards Ratio Sensitivity Analysis**

| Quartiles | Q1 | Q2 | Q3 | Q4 | Q4 vs. Q1 p-value |
| --- | --- | --- | --- | --- | --- |
| BMI (n) | 2456 | 2470 | 2465 | 2470 |  |
| AF 1000 p-y^1^ | 7.74 | 9.22 | 10.1 | 14.3 |  |
| BMI (median) kg/m^2 | 22.1 | 24.9 | 27.0 | 30.7 |  |
| HR-3^2^ | 1 | 1.2 (0.95 - 1.32) | 1.2 (1.04 - 1.4) | 1.76 (1.50 - 2.06) | < 0.001 |
| WC (n) | 2334 | 2491 | 2301 | 2735 |  |
| AF 1000 p-y^1^ | 7.49 | 9.17 | 10.2 | 14.1 |  |
| WC (median) cm | 81.4 | 89.6 | 95.4 | 106.1 |  |
| HR-3^2^ | 1 | 1.12 (0.99 - 1.38) | 1.30 (1.11 - 1.54) | 1.74 (1.48 - 2.04) | < 0.001 |
| WHR (n) | 2451 | 2455 | 2477 | 2478 |  |
| AF 1000 p-y^1^ | 9.22 | 10.1 | 9.97 | 12.0 |  |
| WHR (median) | 0.87 | 0.92 | 0.96 | 1.02 |  |
| HR-3^2^ | 1 | 1.11 (0.95 - 1.29) | 1.07 (0.918 - 1.25) | 1.34 (1.15 - 1.57) |  |
| WHtR (n) | 2461 | 2453 | 2476 | 2471 |  |
| AF 1000 p-y^1^ | 8.12 | 9.11 | 10.2 | 14.1 |  |
| WHtR (median) | 0.46 | 0.51 | 0.54 | 0.61 |  |
| HR-3^2^ | 1 | 1.01 (0.86 - 1.19) | 1.13 (0.97 - 1.33) | 1.44 (1.23 - 1.69) |  |
| BF % (n) | 1909 | 2333 | 3151 | 2468 |  |
| AF 1000 p-y^1^ | 9.20 | 8.97 | 10.7 | 12.0 |  |
| BF % (median) | 14.2 | 18.0 | 21.4 | 27.2 |  |
| HR-3 | 1 | 0.94 (0.79 - 1.11) | 1.10 (0.94 - 1.29) | 1.20 (1.02 - 1.42) | 0.03 |
| Weight (n) | 2158 | 2737 | 2470 | 2496 |  |
| AF 1000 p-y^2^ | 7.23 | 9.13 | 11,0 | 13.7 |  |
| Weight (median) kg | 66.8 | 76.7 | 84.2 | 97.3 |  |
| HR-3 | 1 | 1.24 (1.05 - 1.47) | 1.52 (1.29 - 1.81) | 2.02 (1.71 - 2.39) | < 0.001 |
| Height (n) | 2199 | 2174 | 2817 | 2671 |  |
| AF 1000 p-y^1^ | 9.40 | 9.48 | 11.1 | 10.9 |  |
| Height (median) cm | 74.4 | 79.1 | 82.7 | 88.4 |  |
| HR-3^2^ | 1 | 1.07 (0.91 - 1.26) | 1.35 (1.16 - 1.57) | 1.53 (1.31 - 1.79) |  |
| **^Table S6A – Incidence of Atrial Fibrillation with no prior myocardial infarction or heart failure in relation to anthropometric measures in men^**  ^1^measured in 1000-person years; ^2^Cox-regression Hazard Ratio (HR) adjusted for age (95 % CI); ^2^HR-3 adjusted for HR-2 plus alcohol consumption, low education, marital status and immigrant status (95 % CI). AF = Atrial Fibrillation; BMI = body mass index; WC = waist circumference; WHR = waist-hip ratio; WHtR = waist-height ratio; BF % = body-fat percentage; CI = confidence interval | | | | | |

**Table S6B – Hazards Ratio Sensitivity analysis**

| Quartiles | Q1 | Q2 | Q3 | Q4 | Q4 vs. Q1 p-value |
| --- | --- | --- | --- | --- | --- |
| BMI (n) | 3915 | 3987 | 3910 | 4246 |  |
| AF 1000 p-y^1^ | 4.14 | 5.14 | 6.55 | 8.78 |  |
| BMI (median) kg/m^2 | 20.8 | 23.6 | 26.0 | 31.1 |  |
| HR-3^2^ | 1 | 1.10 (0.94 - 1.28) | 1.21 (1.04 - 1.41) | 1.47 (1.26 - 1.71) | < 0.001 |
| WC (n) | 3738 | 3806 | 4255 | 4259 |  |
| AF 1000 p-y^1^ | 4.02 | 4.91 | 6.22 | 9.10 |  |
| WC (median) cm | 65.9 | 72.5 | 78.7 | 91.4 |  |
| HR-3^2^ | 1 | 1.09 (0.92 - 1.28) | 1.29 (1.10 - 1.52) | 1.58 (1.34 - 1.85) | < 0.001 |
| WHR (n) | 4009 | 4019 | 4006 | 4024 |  |
| AF 1000 p-y^1^ | 5.39 | 5.31 | 5.91 | 7.98 |  |
| WHR (median) | 0.73 | 0.77 | 0.81 | 0.86 |  |
| HR-3^2^ | 1 | 1.03 (0.89 - 1.19) | 1.07 (0.93 - 1.24) | 1.30 (1.13 - 1.49) | < 0.001 |
| WHtR (n) | 3108 | 4487 | 3706 | 4757 |  |
| AF 1000 p-y^1^ | 3.95 | 5.24 | 6.59 | 8.95 |  |
| WHtR (median) | 0.40 | 0.45 | 0.48 | 0.57 |  |
| HR-3^2^ | 1 | 1.09 (0.93 - 1.27) | 1.20 (1.03 - 1.41) | 1.35 (1.15 - 1.58) | < 0.001 |
| BF % (n) | 3987 | 4029 | 3991 | 4051 |  |
| AF 1000 p-y^1^ | 3.81 | 5.12 | 6.07 | 8.74 |  |
| BF % (median) | 23.5 | 28.6 | 32.0 | 36.4 |  |
| HR-3^2^ | 1 | 1.20 (1.01 - 1.42) | 1.18 (0.99 1.40) | 1.46 (1.24 - 1.73) | < 0.001 |
| Weight (n) | 3497 | 4224 | 3989 | 4348 |  |
| AF 1000 p-y^1^ | 4.18 | 4.88 | 6.31 | 8.82 |  |
| Weight (median) kg | 54.7 | 62.6 | 69.2 | 83.0 |  |
| HR-3^2^ | 1 | 1.16 (0.99 - 1.37) | 1.50 (1.28 - 1.74) | 1.91 (1.64 - 2.22) | < 0.001 |
| Height (n) | 4002 | 4025 | 4015 | 4016 |  |
| AF 1000 p-y^1^ | 6.11 | 5.88 | 5.95 | 6.50 |  |
| Height (median) cm | 156 | 162 | 165 | 171 |  |
| HR-3^2^ | 1 | 1.18 (1.02 - 1.35) | 1.36 (1.18 - 1.57) | 1.94 (1.69 - 2.23) | < 0.001 |
| **^Table S6B – Incidence of Atrial Fibrillation with no prior myocardial infarction or heart failure in Relation to anthropometric measures in women^**  ^1^measured in 1000-person years; ^2^Cox-regression Hazard Ratio (HR) adjusted for age (95 % CI); ^2^HR-3 adjusted for HR-2 plus alcohol consumption, low education, marital status and immigrant status (95 % CI). AF = Atrial Fibrillation; BMI = body mass index; WC = waist circumference; WHR = waist-hip ratio; WHtR = waist-height ratio; BF % = body-fat percentage; CI = confidence interval | | | | | |
